# Erysipelas: complement system and SIRS

**DOI:** 10.1186/cc11748

**Published:** 2012-11-14

**Authors:** O Chernyshev, A Petrov, A Shatil, N Serebryanaya, N Bubnova

**Affiliations:** 1 City Hospital of St. George, Saint-Petersburg, Russia; 2 State Research Institute of Highly Pure Biopreparations, Saint-Petersburg, Russia; 3 Saint-Petersburg State Medical University of I.P. Pavlov, Saint-Petersburg, Russia

## Background

Erysipelas in some patients can be complicated with SIRS and sepsis, even with septic shock that may be explained by the hyperactivity of the complement system. The complement system in erysipelas has not been explored thoroughly. Previously a reduction of the general hemolytic activity of different complement components was reported. The study of the complement system is absolutely required to determine the functional state of the immune system and inflammation process in erysipelas.

## Methods

Plasma samples from 38 patients with different clinical erysipelas forms (two primary erysipelas, 12 recurrent erysipelas) were obtained in 2010. The erythematous form was diagnosed in 10 patients with primary infection and in four patients with recurrent infection, the erythematous-hemorrhagic form in eight and seven patients, and the bullous-hemorrhagic form in seven and two patients accordingly. Blood samples were taken on day 1 (admission to the hospital) and on days 7 to 10 (on discharge from the hospital). The evaluation of the concentration of the complement components was performed by commercial solid-phase ELISA. The components studied were: C1-inhibitor (C1-INH) (μg/ml), C4 (mg/ml), C3 (mg/ml), C5 (μg/ml), anafilatoxins C3a (ng/ml) and C5a (ng/ml), and a hydrolysed form of the C3 component C3[H_2_O] (mg/ml and % of total C3) that is triggered by the alternative pathway.

## Results

There were significantly increased concentrations of anafilatoxins C3a and C5a, C1-INH and C3[H_2_O] in erysipelas patients compared with healthy individuals. Correlations were found between serum concentrations of C4 and C3 (*r *= 0.89; *P *< 0.01) and anafilatoxins C3a and C5a (*r *= 0.55; *P *< 0.01). Significant differences were found in different clinical erysipelas forms: patients with primary erysipelas (erythematous form) had significantly higher concentrations of anafilatoxin C3a than patients with the more severe bullous-hemorrhagic form (*P *= 0.02). These data support the hypothesis that C3a has regulatory anti-inflammatory features that make it differ from the definitely proinflammatory C5a component. The portion of C3[H_2_O] was significantly lower in the group of patients with recurrent erysipelas (erythematous form) than in the combined group of erythematous-hemorrhagic and bullous-hemorrhagic erysipelas forms (*P *= 0.04). This suggests that spontaneous complement activation via an alternative pathway is less frequent in patients with mild erysipelas forms. After the treatment period we observed a significantly decreased percentage of C3[H_2_O] in patients with primary (bullous-hemorrhagic form) erysipelas (*P *= 0.02) and in patients with recurrent erysipelas (erythematous-hemorrhagic and bullous-hemorrhagic forms) erysipelas (*P *= 0.02). This proves modern knowledge about decreasing complement activity during reduction of inflammation.

## Conclusion

It was shown that the complement system is significantly activated in erysipelas patients due to increased concentrations of C3a (~3 to 5 times), C5a (~1.5 times), C1-INH (~1.5 times) and % C3[H_2_O] (~100 times) compared with healthy individuals. We suggest that investigation of the complement activity may allow one to improve the estimation of inflammation severity, and predict development of SIRS and effectiveness of therapy.

